# Developing an Animal Welfare Assessment Protocol for Cows in Extensive Beef Cow–Calf Systems in New Zealand. Part 1: Assessing the Feasibility of Identified Animal Welfare Assessment Measures

**DOI:** 10.3390/ani10091597

**Published:** 2020-09-08

**Authors:** Y. Baby Kaurivi, Richard Laven, Rebecca Hickson, Tim Parkinson, Kevin Stafford

**Affiliations:** 1 School of Veterinary Medicine, Massey University, Private Bag 11 222, Palmerston North 4442, New Zealand; r.laven@massey.ac.nz (R.L.); t.j.parkinson@massey.ac.nz (T.P.); 2 School of Agriculture and Environmental Management, Massey University, Private Bag 11 222, Palmerston North 4442, New Zealand; R.Hickson@massey.ac.nz (R.H.); K.J.Stafford@massey.ac.nz (K.S.)

**Keywords:** welfare assessment, beef cows, extensive systems, New Zealand

## Abstract

**Simple Summary:**

It is not feasible to transfer animal welfare assessment protocols developed for intensive systems to extensive systems or from rangeland- to pasture-based cattle because each system needs a different protocol. In a previous study, we combined selected measures from the Welfare Quality protocol for beef cattle and the UC Davis Cow-Calf Health and Handling protocol with additional measures specific to New Zealand to create a welfare assessment protocol for pasture-based cow–calf systems that had 50 measures. In this study, the feasibility of this protocol was assessed during routine yardings of cows and a questionnaire. Individual measures that were deemed unsuitable were eliminated or modified. At the end of the process, a robust, achievable protocol with 32 measures for use on pasture-based extensive cow–calf farms was created.

**Abstract:**

Potential measures suitable for assessing welfare in pasture-based beef cow–calf systems in New Zealand were identified from Welfare Quality and UC Davis Cow-Calf protocols. These were trialled on a single farm and a potential protocol of 50 measures created. The aim of this study was to assess the feasibility of the measures included in this protocol on multiple farms in order, to develop a credible animal welfare assessment protocol for pasture-based cow–calf farms systems in New Zealand. The assessment protocol was trialled on 25 farms over two visits and took a total of 2.5 h over both visits for a 100-cow herd. The first visit in autumn included an animal welfare assessment of 3366 cows during pregnancy scanning, while the second visit in winter included a questionnaire-guided interview to assess cattle management and health, and a farm resource evaluation. Through a process of eliminating unsuitable measures, adjustments of modifiable measures and retaining feasible measures, a protocol with 32 measures was created. The application of the protocol on the farms showed that not all measures are feasible for on-farm assessment, and categorisation of identified animal welfare measures into scores that indicate a threshold of acceptable and non-acceptable welfare standards is necessary.

## 1. Introduction

Good animal welfare is increasingly recognised as an important component in the trade of farm animals and their products [1,2]. This has resulted in the development of welfare assessment protocols for specific production systems [3] which support trade, farm assurance, and product labelling. Properly planned assessments can identify risk factors for poor welfare, aid in the development of interventions, and be used to monitor and evaluate changes in practice [2,4,5].

Most protocols are based on a mixture of animal- and input-based assessments [6,7], along with assessments of stockpersonship [8]. Almost all widely used protocols are directed at intensive systems, due to the perception, especially in Europe, that confined systems lack the ‘naturalness’ [1] of pasture-based systems. It is not possible to just transfer protocols developed for intensive systems to extensive systems. For example, measures such as hock lesions and rising restrictions, both of which are essential measures in welfare assessments of housed cattle, are generally irrelevant to New Zealand because cattle are not normally housed [9]. This means that to assess the welfare of beef cattle in New Zealand, the assessment must be designed for the extensive pasture-based beef system which predominates there.

In New Zealand, most farms that have breeding cows are on hill or high country and are also sheep farms. On many farms, the main purpose of beef cattle is to maintain good-quality pasture for the sheep. On most farms grazing policies are not consistent throughout the year with paddocks being set stocked in spring but rotationally grazed in the rest of the year. The predominant cow breeds are Angus and Hereford and their crosses. On commercial beef farms, herd sizes range from 50–60 breeding cows to over 500. Cows calve in spring (August to October) with steers and non-replacement heifers sold in September to November [10].

A protocol that combined selected measures from the Welfare Quality beef cow protocol [7] and the UC Davis Cow-Calf Health and Handling protocol [11] with additional measures specific to New Zealand was recently created [12]. This protocol required testing for practicability and feasibility across multiple beef farms before it can be used more widely. The aim of this study was to evaluate the practicability and feasibility using the protocol for welfare assessment on 25 cow–calf farms during routine pregnancy testing. This aim was achieved through the application of measures on commercial farms and making judgements as to whether they were feasible to record, required modification, were already adequately captured by another measure or were not practical to measure in a commercial setting. The scope of the current assessment protocol included replacement heifers and cows at pregnancy testing and excluded calves, steers and bulls.

## 2. Materials and Methods

### 2.1. Farms

The study was carried out on 25 extensive pasture-based cow–calf herds in the Waikato region of the North Island of New Zealand. This was a sample of the clients of a veterinary practice (VetEnt, Te Awamutu, New Zealand) who used that practice for routine pregnancy diagnosis in autumn. Herd details are summarised in Table 1. All farms were mixed sheep and beef enterprises and used rotational grazing as their main means of feeding cattle, with supplementary hay or silage fed during winter. No housing or off-pasture feeding was used.

### 2.2. Welfare Assessment and Data Collection

The protocol development process involved two phases. Phase 1 took place in March/April 2018 (autumn) at the time of pregnancy diagnosis and Phase 2 in winter 2018 (July) at a time when their natural environment is likely to be at its worst. Following the identification of suitable measures at one farm by three of the authors and an experienced technician where inter observer reliability was assured [12], the assessments in Phases 1 and 2 of the current study were undertaken by only one of the authors.

Phase 1

All assessments were undertaken when cattle were brought in for pregnancy diagnosis. Farms varied in how cattle were brought in for pregnancy diagnosis. Some cows were brought in from a nearby paddock when the veterinarians arrived, some were put in the holding pen in the early morning for pregnancy testing in the late morning or afternoon, and some were kept overnight in holding pens. All but one farm used herding dogs and bikes to bring the cows in (one farm had no dogs). Pregnancy diagnosis was done in a full race, using ultrasound with occasional manual examination for confirmation). Once all the cows in the race were tested, cows were let out into the holding pen or paddock, and the race refilled. Observations were made of cows in the holding pens and in the race during pregnancy diagnosis. Whilst in the holding pens, the cows were observed for physical health, body condition, rumen-fill, and behaviour. Video recordings (2 × 15–20 min) were also made of the herd in the holding pens for qualitative behavioural evaluations and as a back-up tool and were transcribed at the end of each farm visit.

Observations of cows in the race included the interaction between the stockperson and the cows, as the latter entered, were handled and exited the race, and the effects of race design (including the transition from holding pen to race) upon cow behaviour. As animals exited the race, their exit speed (running or walking), whether they fell or stumbled, and lameness signs were all recorded. The design and quality of handling facilities were concurrently assessed. Information was collected on yard accessibility and yard design, with emphasis on flooring, shape and size of forcing pens, race structure and the presence/absence of solid-sided walls. The position of the observer was constrained by the design of the race but observations of cows exiting the race were made by the assessor standing as close to the exit as possible without interfering with cow flow. All other observations were made in single-file races. All animal-based assessments were made in the race, following the veterinarian who was doing pregnancy diagnosis. In some cases, the veterinarian started at the front of the race, others at the back. As many animals were assessed per race as possible without slowing the pregnancy diagnosis process, with a target of observing more than 50% of cows in a herd (Table 1). All assessments took place between 9:00 and 16:00. Phase 1 visits typically lasted 1 h per 100 cows in the herd.

Phase 2

A questionnaire-guided interview (see Appendix A) was conducted with the farm manager to assess health and management of each herd in the last 12 months. The general management and key health aspects on the farm (dehorning/disbudding, castration, vaccination, diseases or disease symptoms seen in cattle, cattle deaths, access and type of water supply, feed/pasture condition, wintering practices) were recorded.

A visit to observe cows at pasture (in at least one paddock per farm) was made to assess provision of grazing, access to water and shade in the paddocks, pasture hazards (e.g., steep hills and sinkholes), and to get a general overview of cow body condition. In addition, the flight zone was assessed when the assessor (with the farmer) moved closer to where the cows were in the paddock. Phase 2 visits typically lasted 1.5 h.

See Table 2, Table 3 and Table 4 for details on how each measure was recorded.

### 2.3. Data Analysis

Data were analysed using SPSS version 24 (IBM). Spearman’s rank correlation was used to identify measures with a strong association (ρ ≥ 0.8).

Following the visits, after consideration of farms variations and data analysis, the feasibility of the individual measures was divided by four of the authors into:

- Measures that were retained unchanged in final protocol;
- Measures that were not feasible on all farms but were considered necessary and suitable for keeping in an adjusted form in the final protocol;
- Measures that were significantly (ρ ≥ 0.8) correlated with other measures, which could be rationalised into a single measure;
- Measures that were not feasible across all farms and or which were deemed to be unnecessary or unsuitable. These measures were removed from the final protocol.

Specific reasons for categorising each measure in these four groups are described in the results section (Table 2, Table 3 and Table 4). Broadly, the exclusion or modification of measures from the protocol was mainly due to the difficulty of measurements across all farms, questionable or unclear welfare implications to the production system, time and space limitation, measure requiring specialized assessments and adjustments of location of measuring to where it was more achievable.

## 3. Results

A total of 4956 cows were presented for pregnancy diagnosis, with yard observations made on 3366 animals (Table 1). Measures included in the final protocol without adjustment are shown in Table 2. Twelve measures were modified before inclusion in the final protocol. These measures are shown in Table 3, along with the reason for modification and the suggested modification. Correlations of animal welfare measures with a Spearman’s correlation coefficient of ρ ≥ 0.5 and confidence interval are shown in Table 5. Only three measures were highly (ρ > 0.8) correlated: dirty tails and dirty hind quarters (ρ = 0.85), dirty tails and dirty flanks (ρ = 0.86), and dirty hind quarters and dirty flank (ρ = 0.89). These measures were combined for welfare assessment into one measure of “dirtiness” by averaging the three measures per farm. Other significant correlations were between; thin cows and poor rumen fill (ρ = 0.76), dirty tail and diarrhoea (ρ = 0.75), hit and noise of handlers (ρ = 0.73), fall and fearful (ρ = 0.64) and yarding frequency/year and fearful (ρ = 0.50). Eight measures were excluded as it was not feasible to assess them during a pregnancy diagnosis visit and they were not necessary to keep in an adjusted form. These measures are shown in Table 4, along with rationale for their removal.

**Figure 1:**
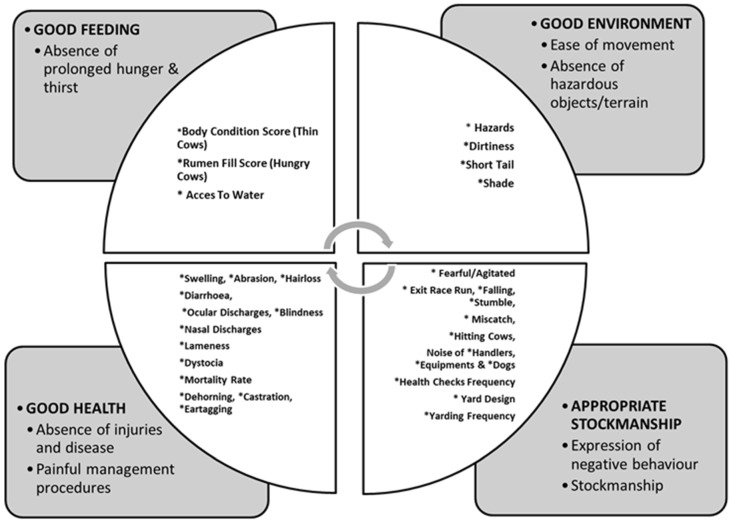
Summary of the 32 measures determined as being suitable for inclusion in a welfare assessment protocol for extensively reared pasture-based beef cattle in New Zealand.

The final protocol included 32 measures, as summarised in Figure 1. Three measures were related to good feeding, four to appropriate environment, 16 to health measures and 13 to stockpersonship. The data collected (summary statistics) will be reported and discussed in Part 2 (Kaurivi submitted).

## 4. Discussion

The aim of this study was to create a protocol in which most of the animal-based measures could be made in cattle that were being handled for purposes other than the assessment itself. To this end, the study evaluated the feasibility of applying 50 potential measures of welfare [11] in extensively managed beef cattle during routine yarding for pregnancy diagnosis and during a paddock observation; on the basis that there is limited value in recording measures that require a specific separate examination [9]. Of the 50 initial measures, 8 were excluded because it was not feasible to collect them across all farms during yarding and it was not worth routinely recording the prevalence of broken tails but if they are observed their presence should be noted. Three highly correlated measures (ρ > 0.8) were amalgamated into one combined measure. Avoidance distance was the only animal-based assessment made at pasture as it was not feasible to observe this during yarding. However, it is known that this can be difficult to assess in cows at pasture [15]. This proved to be the case in this study as, on many farms, cows associated the arrival of people with being moved to fresh grazing and so this measure was deemed unfeasible.

Moreover, the process of making the observations should not, of itself, impinge upon the welfare of the cattle. Thus, beef cattle that are managed in extensive systems are infrequently yarded and probably associate yarding with bad experiences and become stressed in them [16,17,18]. Conversely, cattle that are frequently yarded show fewer signs of agitation, such as vocalisation and stumbling [8] in handling facilities [17,19] than do those that are seldom yarded. Thus, the number of yardings is itself a determinant of the welfare of the cows, so setting thresholds of yarding of cows per year (e.g., 3–6 times per year in this study) should reflect the impact of yarding on the welfare of the animals. Further research is required to validate these thresholds and determine the effect of handling frequency itself on welfare of beef cows in New Zealand.

Measures should not be excluded from a protocol simply because they are absent, as their absence does demonstrate the lack of a welfare problem. Broken tail was excluded because it was not observed on any farm and farmers were not observed twisting or manipulating cows’ tails (probably the main cause of broken tails outside of accidental injury [20]). However, in this case, the exclusion does not mean it will not be observed, as broken tails can be easily observed if they are common.

Many measures can potentially be used to assess the welfare of cattle at pasture (e.g., [21]). However, if a measure is to be used in an assessment programme it must be repeatable and comparable across operations in similar production systems [5,22] and it must be achievable within a limited time frame alongside other assessments [5,6,23]. This requirement to undertake most of the assessments alongside a routine handling process has the benefit that the measures of stock handling reflect a real process but has the disadvantage that some animal-based measures are difficult or impossible to achieve. Hence, of the measures that were removed from the final protocol in this study, most were excluded primarily because they were difficult to assess in a single-file race from an elevated platform next to the race. These include recording baulking and assessing hoof problems and udder dirtiness. Similarly, the requirement that cattle were going through a routine handling process meant that, even with video cameras, complex behaviours could not be assessed as there was insufficient space and time [24] in the collecting pens for cattle to show such behaviours. Additionally, management practices during the handling limited the applicability of some measures. This was particularly so for vocalisation which can be a useful measure of stockpersonship, but on most farms was simply a measure of separation anxiety of cows from calves [25]. The requirement for measures to be repeatable and comparable across operations means that this protocol need to be tested on more farms by more assessors to validate whether measures are repeatable across assessors and whether differences between farms affect feasibility.

Of the original 50, 11 measures required modification based on the feasibility of making those observations during routine yarding. Many of these modifications were relatively trivial, commonly involving changing the position of the observer whilst the assessments were being made, and usually dictated by the design of the handling facilities. Thus, it proved more practical to assess mis-catching of cows with gates on any part of the body in the race instead of the head bale; hitting of cows in forcing pens versus in the race, and observing falling down while drafted into the race rather than only on exiting. The assessment of measures such as running, stumbling and lameness designed for cows exiting the race to paddocks also required adjustments to include those exited from holding pens to paddocks.

Conversely, other measures proved more amenable to converting to a categorical basis than originally envisaged. The provision of water is seen as a fundamental criterion of animal welfare, but in New Zealand, where it is sourced naturally, water is abundant, and is universally provided to cattle in water troughs, so devising an appropriate assessment criterion was challenging. The final criterion, namely, whether there was water available within 500 m, was based on the literature showing that 250 m is the optimum distance for cows to walk to water in terms of the ideal distance for optimum productivity [26,27]. Likewise, provision of shade is a critical criterion in terms of maintaining an animal thermal comfort [15,28]; however, in the present study, it was rarely an issue given the presence of trees and terrain shadows in paddocks on the beef farms under study.

It was difficult to make a useful assessment of dirtiness as a criterion of animal welfare. Dirt over the flank, hind and tail were highly correlated (ρ > 0.8) with each other, so were converted into a combined measure in the final protocol, as cows which are dirty in one place are usually dirty in the others. Dirtiness can be a useful measure of welfare in intensive dairy cows [29,30] and housed beef cows [31,32] where faecal contamination of the environment have a higher chance of udder, hoof and skin infections. However, it is probably not meaningful to equate the faecal contamination of housed cattle with the muddiness of pastured cattle [9,33]. The impact of this measure on the welfare of pastured cattle is, at best, ambiguous and may really only be of significance in terms of the risk of meat contamination when cattle are slaughtered [34]. Assessment of the presence of diarrhoea, again an important criterion for housed cattle, was also difficult in the present study. In New Zealand, most pasture-fed beef cows have watery faeces for most of the year, leading to a high level of faecal soiling [35,36]. Even in rangeland systems, the importance of diarrhoea and faecal soiling as measures of welfare have been questioned: for example, Simon [22] observed a high rate of faecal soiling at most California beef cattle ranches. Faecal soiling of the tail may result in “short tails”. Short tails are tails that have been sloughed off as a result of constriction of blood supply to the distal tails by hardened faecal rings [12,37]. Since this undoubtedly causes pain to the animal, short tail is an important indicator of adverse effects from faecal soiling. Measures that require complicated assessment need feasibility evaluation to be included in assessment protocols. For example, disease history requires considerable detail to establish the impact of a disease on cattle welfare. Such detail was not available on most farms. Similar restrictions meant that we recorded overall mortality rate and there was no differentiation than between voluntary and involuntary culling [38]. Such a differentiation would have provided further information in addition to the mortality rate, by identifying the rate at which health problems necessitated the culling of cows, but it was not possible in this study.

Nonetheless, despite the aforegoing caveats, most of the measures proposed by Kaurivi [12] proved to be both feasible and useful. The use of body condition score (BCS) as a means of identifying the adequacy of feeding is, of course, well documented. In the present study, BCS was assessed against the New Zealand scale of 1–10 [13], with scale of 1–4 taken as thin cows. However, from a production point, BCS 4 could be acceptable [39], but from an animal welfare perspective may be regarded as low and unacceptable. Moreover, there was a strong positive correlation (ρ = 0.76) between BCS (thin cows) and poor rumen fill scores (RFS). However, although both measures are good indicators of nutritional status of cows, they provide different information: BCS about the medium-term nutritional status of the animals, RFS about their recent intake [14,40]. Therefore, for large extensive beef farms where cattle are commonly yarded the day before any management procedure, the use of RFS by itself as a valid indicator of welfare is questionable. Nonetheless, poor RFS over a period could be a good indicator for farmers to monitor specific individual animals closely, adjust the herd’s feed intake, or investigate problems that could be causing the prolonged poor RFS [41]. Taken together, such arguments justified retaining RFS as well as BCS in the final protocol.

Although the assessment of body injuries was regarded as feasible, setting the thresholds at which they would be regarded as abnormal required careful consideration. Various criteria have been set for skin alterations, varying from >5 cm [42] and >2 cm [22] to >1 cm in the present study. Generally, such determinations represent a compromise between what is likely to represent a negative effect upon the animal and what can be repeatedly assessed within the constraints of the situation in which the observations are made. A similar situation might arise in ranking the severity of body integument alterations, differentiating animals or farms with small or few lesions on the body of animals from those with big or many severe lesions [43]. This was not considered in this study for practical reasons.

Likewise, careful consideration was also given to categorise measures (e.g., stockpersonship measures) into three tiers to reflect severity. In the evaluation of noise at the yards, subjective thresholds were used to indicate severity, i.e., no noise to moderate/minimal noise versus very noisy handlers/ equipment and no dogs at the yards *vs* quiet to constant noisy dogs. The grading developed in the present study aligns with Petherick [44] that sudden exposure to noise is stressful to cattle and may result in stampeding. A similar conclusion from Waynert [45] was that the reduction of metal clicking, and human shouting could reduce fear in cattle. Cattle respond to vocalisations of other species [46,47] and noisy dogs are likely to affect the behaviour of cattle [48], e.g., agitation and running on exit from race. Hence, the subjective weighing to indicate severity of this measure was necessary for a welfare assessment protocol.

The final assessment related to the design of yards and handling facilities. The difficulty in achieving optimal design is reflected in the many recommendations that are extant for facility design and handling infrastructure [22,49,50,51]. Consequently, various farmers opt for different yard designs, resulting in variable effectiveness of cattle flow. In the present study, it was clear that facilities fell into three categories: those that were associated with easy flow of cattle, those whose cattle flow could be improved with minor adjustments, and those that would require major remediation to allow effective cattle flow.

## 5. Conclusions

This study has taken a series of measures that appear intrinsically suitable to the assessment of welfare of beef cows and has evaluated the feasibility of assessing those measures in cows during routine yardings and questionnaire on cattle and farm management. Whilst the 25 farms in this study do not necessarily represent the welfare conditions of beef cows throughout New Zealand and inter observer reliability was not assured, they were suitable for developing and validating feasible welfare measures for an assessment protocol. Through elimination of measures that were unsuitable for use, or required modification for use under those circumstances, or which yielded information that proved to be of little value, a robust, achievable protocol of 32 measures has been developed for use on pasture-based extensive cow–calf farms in New Zealand. The proposed protocol is envisioned as being suitable for use at farm level for benchmarking and certification of welfare standards on farms. Further research is required to establish this protocol with more observers and more farms across New Zealand.

The next immediate step of the research is to categorise identified feasible animal welfare measures into scores that indicate a threshold of acceptable versus unacceptable welfare standards, in order to explore the development of an animal welfare assessment protocol for extensive beef systems in New Zealand. These thresholds will provide indicators to farmers and farm advisors regarding the levels at which intervention and remediation is required. This will be addressed in a companion paper to this one (https://www.mdpi.com/2076-2615/10/9/1592).

## Figures and Tables

**Table 1:** Details of farms, cow herds presented for pregnancy testing and percentage of cows assessed for animal-based welfare measures in the study.

	Topography	Breed	Age	Total Herd	Number Assessed	% Assessed
Farm 1	Hill	Angus	Mixed	253	176	70
Farm 2	Hill	Angus and Charolais	Mixed	410	209	51
Farm 3	Hill	Hereford	Heifers	33	25	76
Farm 4	Hill	Devon and Angus	Mixed	97	67	69
Farm 5	Hill	Angus and Simmental	Mixed	123	78	63
Farm 6	Hill	Hereford	Mixed	212	163	77
Farm 7	Hills rolling	Belted Galloway	Young	18	18	100
Farm 8	Hills rolling	Angus	Mixed	318	272	86
Farm 9	Hills	Angus × Simmental	Mixed	305	239	78
Farm 10	Hills	Angus and Hereford	Mixed	241	180	75
Farm 11	Hills	Angus and Friesian	Mixed	103	101	98
Farm 12	Hills	Angus	Mixed	154	133	86
Farm 13	High	Angus and Devon	Mixed	145	113	78
Farm 14	Hill	Angus	Young	162	133	82
Farm 15	Flat to rolling	Angus × Hereford	Mixed	96	61	64
Farm 16	Flat to rolling	Angus × dairy breed	Mixed	30	28	93
Farm 17	Flat to rolling	Hereford	Mixed	81	52	64
Farm 18	High	Angus	Mixed	293	155	53
Farm 19	High	Angus	Mixed	98	62	63
Farm 20	High	Angus and crosses	Mixed	464	308	66
Farm 21	Hill to high	Angus and crosses	Mixed	174	124	71
Farm 22	Hill	Angus	Mixed	232	137	59
Farm 23	Hill rolling	Angus and crosses	Mixed	541	304	56
Farm 24	Hill	Mixed Angus with Devon	Mixed	306	171	56
Farm 25	High	Angus and crosses	Mixed	67	57	85
			Total	4956	3366	
‘Mixed’: heifers and cows			Average	196	135	73
‘Young’: heifers and yearlings			Median	162	133	71

**Table 2:** Measures assessed as feasible for inclusion in the final protocol without change (observations in and around the race were done in Phase 1, and questionnaire assessments in Phase 2).

Principle	Welfare Criteria	Welfare Measures	Method of Assessment (Observation in and Around Race Unless Otherwise Stated)
Good feeding	Absence of hunger	Body condition Score	% thin cows in the herd, based on score ≤4/10 on 1–10 scale [13].
		Rumen Fill	% of cows with hollow/empty rumen [14]
Appropriate Environment	Thermal comfort	Shade	Subjective assessment of shade in the paddocks (presence of trees, shrubs, galleys, human-made canopies) as enough or insufficient.
	Comfort around resting place	Short tail	% of cows with short tail (sloughed-off tails)
Good Health	Absence of injuries/physical impairment	Abrasions	% of cows with abrasions/fresh scratches or cuts extending >1 cm.
		Swelling	% of cows with swellings of >1 cm in diameter.
		Hair loss/hairless	% of cows with hairless patches of >1 cm.
	Absence of disease and pain	Blindness	% of cows with “affected eye (s)” by visual assessment and/or testing with hand.
		Ocular discharges	% of cows with evidence of ocular discharges extending 2 cm.
		Nasal discharges	% of cows with evidence of nasal discharges extending 2 cm.
		Dystocia	% as reported by the famers during questionnaire-guided interview.
	Absence of pain from management procedures	Ear tagging, Castration, Disbudding	Record age and use of local anaesthetic during questionnaire-guided interview.
Stockpersonship	Animal handling stockpersonship and resource-based measures	Noise of handlers	Subjective categorical assessment of handler noise: (1) none; (2) some but not frequent; (3) loud and repeated shouting.
		Noise of Equipment/machinery	Subjective categorical assessment of noise of equipment: (e.g., race or chute gate) and machinery (e.g., generators): (1) no noise, (2) minor audible noise or (3) unpleasantly noisy.
		Dogs herding cattle and noise around the yard	Categorical subjective assessment: (1) no dogs, (2) quiet dogs or (3) noisy/repeatedly audible dogs.
		Health checks	Record frequency of health checks on cows during winter/pregnancy (in questionnaire)
		Yarding frequency	Record frequency (number of times) of yarding of cows per year (in questionnaire).
		Yard design flow	Subjective categorical assessment: (1) easy movement and flow; (2) effective movement with minor problems (e.g., more gates needed) and (3) poor flow and difficult handling (e.g., forcing pen with too many corners or gate too big).

**Table 3:** Measures included in the protocol after adjustments, including the rationale for change and the changes that were made (observations in and around the race were done in Phase 1, and questionnaire assessments in Phase 2).

Welfare Principles	Measures	Method of Assessment Q: Questionnaire, D: Direct Observation	Reason for Difficulty	Adjustment of Measures and Outcome and Recommendation
Good feeding	Access to water	Q: How far cattle had to walk to access water	Difficult to estimate due to farm terrain.	Adjusted to a categorical measure based on average distance to water source (<250; 250–500 m and >500 m).
Appropriate Environment	Hazards	D: Identify pasture hazards (e.g., steep hills, cliffs, gullies, and sinkholes), and presence of dangerous objects/garbage.	Required a categorical scale.	Adjusted to a 3-point scale: 1; No hazards, 2; ≤two hazards; 3; >2 hazards or animals dying in any hazard.
Good Health	Disease history	Q: Occurrence of diseases of minor (e.g., warts), major (e.g., theileriosis) or variable (e.g., fasciolosis) significance to welfare	There were no common diseases on these farms which can act as proxies for disease control in general (as could, for example, cases of respiratory disease on beef finishing units).	Disease data collection kept in the protocol but not used as part of an individual farm’s assessment (except dystocia and mortality)
	Mortality	Q: % of cows which died on the farm or were culled due to disease or accidents during the last 12 months.	Farmers did not discriminate between cow deaths and all animal deaths. Culling data did not differentiate between voluntary and involuntary.	Numbers of accidental deaths and deaths/slaughter (either on-farm or sent off-farm) due to disease were combined.
	Lameness	D: % of cattle with uneven weight-bearing on a limb that is immediately identifiable and/or obviously shortened stride).	It was often not possible to observe individual cows as they exited the race into a holding pen with other cows.	Adjusted so that lameness was also assessed as cows exited from the holding pen to the paddock.
	Diarrhoea	D: ‘Diarrhoea’ was defined as the % of cows with presence of symmetrical wet or dry patches of faeces below the tail head which were at least the size of a hand.	Watery faeces are extremely common in grass-fed cattle in New Zealand because of the high-water content of pasture, so could not be differentiated from a pathological condition.	Re-categorised as a measure of appropriate environment as “faecal soiling” alongside other related measures (i.e., short tail and dirtiness).
Appropriate Behaviour	Negative behaviour	D: Video recording agonistic behaviours, and signs of agitation or fearfulness	In most holding pens there was insufficient space for cows to display those behaviours.	Video records were not used and replaced by recording only observed fearful/agitated behaviour (i.e., persistent pushing, climbing on others, or trying to climb over the fence/rails) in the race.
Stockpersonship	Running and stumbling	D: Running or stumbling was defined as % of cows taking ≥2 strides at a gait faster than a trot, or their knees/hocks contacted the ground, on exiting the race.	Where cows exited from the race into a holding pen, stumbling or running could not be observed as they were usually moving into a group of cows.	Assessment changed so that stumbling or running when cows exit the holding pen to the paddocks was included.
	Fall	D: Falling was defined to capture % of cows whose torso contacted the ground on exiting the race.	No falling was observed. This may have been due to many farms moving cows into holding pens from the race rather than directly into a paddock.	Replaced by recording cows falling or lying down while in the race and forcing pen.
	Hitting cows	D: The % of individual cows aggressively hit or poked with force or repeatedly while in the race was to be recorded.	Most hitting occurred when drafting cattle into the forcing pen from the holding pens than within the race.	Changed to a subjective categorical observation of hitting of the group rather than the individual cow: no hitting; occasional hitting (≤10% of cows); frequent hitting (>10% of cows) into the forcing pen and race.
	Mis-catching	D: % animals mis-caught in the head gate	The head gate was not used routinely on most farms	Changed to a categorical estimate of the proportion of cows that were mis-caught on any part of the body while gates were closed into or within the race (˂1% versus ≥1% mis-caught).

**Table 4:** Welfare measures removed from the protocol after feasibility testing on 25 Waikato beef farms (observations in and around the race were done in Phase 1, and Questionnaire assessments in Phase 2).

Welfare Principles	Measures	Method of Assessment:	Reasons for Removal
Good Environment	Udder dirtiness	>25% of an udder covered with dirt or manure	Difficulty of observing udders of cows in the race. Dirt on udders is more likely to be mud (low welfare risk) than faeces (high risk).
Good Health	Hoof problems	Presence of overgrown, abnormally shaped or cracked hooves in individual cows.	Difficulty of observing hoof confirmation of cows in the race, or in holding pens (individual assessment in a crowd) or when exiting the race (exiting into a crowd or straight into paddocks). Furthermore, the main link between hoof confirmation and welfare is likely to be its impact on lameness, thus measuring an additional criterion seems unnecessary especially when it would require a specific examination.
	Hampered respiration or coughing	Number of coughs or hampered respirations over 15–20 min for 20 cows in pens (video).	It was not possible to determine whether coughing and related signs were due to disease or to the environment of the yards.
	Broken tails	Observations of abnormal tails (misaligned or broken at the tail head).	Broken tails were not observed in the race nor reported (questionnaire) on any of the farms.
Stockpersonship	Baulking	Cows which refuse to move forward, or which move backwards, when the route is clear in front in the race.	Pregnancy testing was performed with a full race, i.e., all cows were lined up in the race, pregnancy tested and then released all at once. There was thus no opportunity to observe baulking
	Vocalisation	Cows which make an audible sound after restraining but before procedure takes place.	On the study farms vocalisation occurred when the cows were brought in, they either saw calves that had been weaned a month or two earlier or were separated from their calves before the cows were put in the holding pen.
	Flight distance	Cows in a group are approached slowly and distance is estimated when withdrawal start to occur. As this requires that they are free to move, this was assessed during Phase 2 of the study.	On some farms, the cattle could only be observed after they had been drafted with bikes and/or dogs which meant they were agitated before observation. On other farms, the presence of the farmer meant cattle were anticipating being moved to a new break of grass and thus approached the observer rather than moved away. it was therefore not possible to make valid determinations of flight distance
	Herding cattle on motorbikes and quadbikes	Recording those farms where cattle were herded on motorbikes and quadbikes.	All farms used motorbikes or quadbikes to herd cows, and it was not possible to apply criteria to assess the ‘quality’ of their use. Assessing handling in races was therefore considered as a more reliable proxy for assessing herding skills.

**Table 5:** Correlations of animal welfare measures with a Spearman’s correlation coefficient of > 0.5 and confidence interval.

				Confidence Interval	
Welfare Criteria	Correlated Measures		Correlation Coefficient	Lower Bound −1.96	Upper Bound +1.96
**Feeding**	Thin cows	Poor rumen fill	0.76	0.52	0.92
**Environment and “Diarrhoea”**	Dirty tail	Dirty hind	**0.85**	0.73	0.92
	Dirty tail	Dirty flank	**0.86**	0.73	0.93
	Dirty flank	Dirty hind	**0.89**	0.74	0.95
	Dirty tail	Diarrhoea	0.75	0.51	0.87
	Dirty hind	Diarrhoea	0.65	0.36	0.83
	Dirty flank	Diarrhoea	0.6	0.26	0.82
**Stockpersonship**	Hit	Noise Handler	0.73	0.49	0.87
	Yard flow	Noise Handlers	0.62	0.39	0.79
	Fall	Fearful	0.64	0.33	0.80
	Yarding/yr	Fearful	0.50	0.19	0.75
**Stockpersonship and Others**	Stumble	Abortion	0.69	0.21	0.94
	Run	Short tail	0.57	0.20	0.78

Bolded measures had a correlation coefficient of > 0.8.

**Figure A1:**
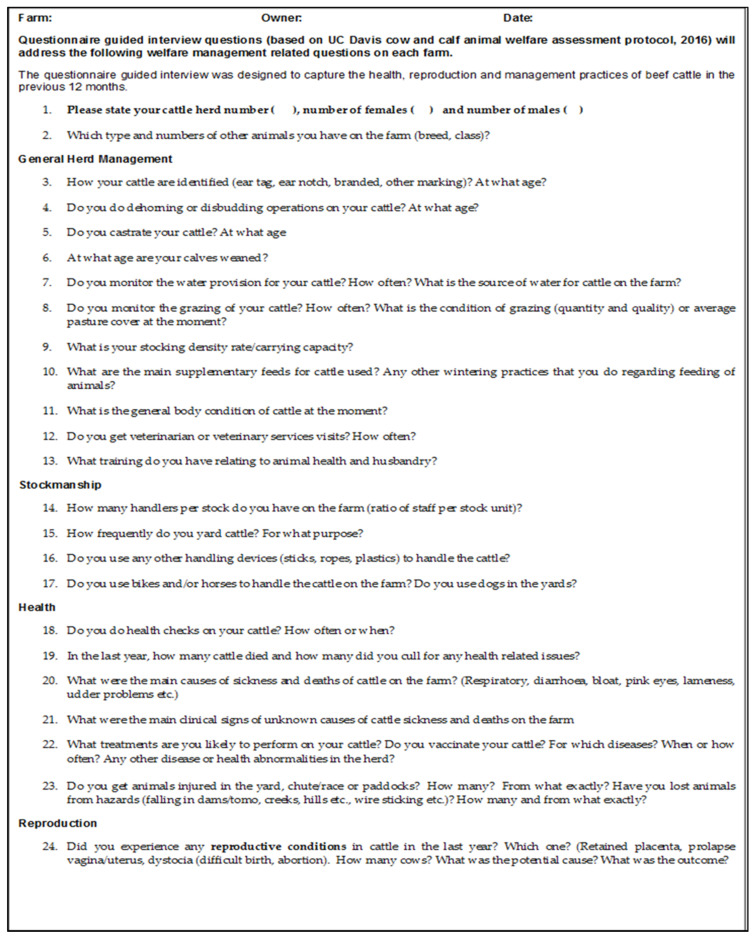
Questionnaire-Guided Interview Questions Used for Welfare Assessment at 25 Waikato Beef Farms.

## References

[1] Fraser D. (2008). Understanding animal welfare. Acta Vet. Scand..

[2] Dunston-Clarke E., Willis R.S., Fleming P.A., Barnes A.L., Miller D.W., Collins T. (2020). Developing an Animal Welfare Assessment Protocol for Livestock Transported by Sea. Animals.

[3] Webster J. (2005). The assessment and implementation of animal welfare: Theory into practice. OIE Rev. Sci. Tech..

[4] Fraser D. (2006). Animal welfare assurance programs in food production: A framework for assessing the options. Anim. Welf..

[5] Knierim U., Winckler C. (2009). On-farm welfare assessment in cattle: Validity, reliability and feasibility issues and future perspectives with special regard to the Welfare Quality^®^ approach. Anim. Welf..

[6] Whay H.R., Main D.C.J., Green L.E., Webster A.J.F. (2003). Assessment of the welfare of dairy cattle using animal-based measurements: Direct observations and investigation of farm records. Vet. Rec..

[7] Welfare Quality (2009). Welfare Quality Assessment Protocol for Cattle. Welfare Quality Assessment Protocol for Cattle (without Veal Calves).

[8] Simon G.E., Hoar B.R., Tucker C.B. (2016). Assessing cow–calf welfare. Part 2: Risk factors for beef cow health and behavior and stockperson handling. J. Anim. Sci..

[9] Laven R.A., Fabian J. (2016). Applying animal-based welfare assessments on New Zealand dairy farms: Feasibility and a comparison with United Kingdom data. N. Z. Vet. J..

[10] Morris S.T., Stafford K. (2017). Beef Cattle Production. Livestock Production in New Zealand.

[11] UC Davis University of California, Davis Cow-Calf Health and Handling Assessment. https://www.ucdcowcalfassessment.com/.

[12] Kaurivi Y.B., Laven R., Hickson R., Stafford K., Parkinson T. (2019). Identification of Suitable Animal Welfare Assessment Measures for Extensive Beef Systems in New Zealand. Agriculture.

[13] Hickson R.E., Morris M.J., Thomson B. (2017). Beef Cow Body Condition Scoring.

[14] Hulsen J. (2005). Cow Signals: A Practical Guide for Dairy Farm Management.

[15] Mancera K.F., Zarza H., de Buen L.L., García A.A.C., Palacios F.M., Galindo F. (2018). Integrating links between tree coverage and cattle welfare in silvopastoral systems evaluation. Agron. Sustain. Dev..

[16] Hemsworth P.H., Coleman G.J. (2010). Human-Animal Interactions And Productivity and Welfare. Human-Livestock Interactions: The Stockperson and the Productivity and Welfare of Intensively Farmed Animals.

[17] Francisco C.L., Cooke R.F., Marques R.S., Mills R.R., Bohnert D.W. (2012). Effects of temperament and acclimation to handling on feedlot performance of bos taurus feeder cattle originated from a rangeland-based cow-calf system. J. Anim. Sci..

[18] Grandin T. (2008). Safe handling of large animals: Part I. IRISH Vet. J..

[19] Cooke R.F., Arthington J.D., Araujo D.B., Lamb G.C. (2009). Effects of acclimation to human interaction on performance, temperament, physiological responses, and pregnancy rates of Brahman-crossbred cows. J. Anim. Sci..

[20] Laven R.A., Jermy M.C. (2020). Measuring the torque required to cause vertebral dislocation in cattle tails. N. Z. Vet. J..

[21] Spigarelli C., Zuliani A., Battini M., Mattiello S., Bovolenta S. (2020). Welfare assessment on pasture: A review on animal-based measures for ruminants. Animals.

[22] Simon G.E., Hoar B.R., Tucker C.B. (2016). Assessing cow–calf welfare. Part 1: Benchmarking beef cow health and behavior, handling; and management, facilities, and producer perspectives. J. Anim. Sci..

[23] Blokhuis H., Veissier I., Jones B., Miele M. (2013). The welfare quality^®^ vision. Improving Farm Animal Welfare Science and Society Working Together: The Welfare Quality Approach.

[24] Barrell G.K. (2019). An Appraisal of Methods for Measuring Welfare of Grazing Ruminants. Front. Vet. Sci..

[25] Pérez-Torres L., Orihuela A., Corro M., Rubio I., Alonso M.A., Galina C.S. (2016). Effects of separation time on behavioral and physiological characteristics of Brahman cows and their calves. Appl. Anim. Behav. Sci..

[26] Hart R.H., Bissio J., Samuel M.J., Waggoner J.W. (1993). Grazing systems, pasture size, and cattle grazing behavior, distribution and gains. J. Range Manag..

[27] Ganskopp D. (2001). Manipulating cattle distribution with salt and water in large arid-land pastures: A GPS/GIS assessment. Appl. Anim. Behav. Sci..

[28] Broom D.M., Galindo F.A., Murgueitio E. (2013). Sustainable, efficient livestock production with high biodiversity and good welfare for animals. Proc. R. Soc. B Biol. Sci..

[29] Logue D.N., Mayne C.S. (2014). Welfare-positive management and nutrition for the dairy herd: A European perspective. Vet. J..

[30] Moreira T.F., Nicolino R.R., Meneses R.M., Fonseca G.V., Rodrigues L.M., Facury Filho E.J., Carvalho A.U. (2019). Risk factors associated with lameness and hoof lesions in pasture-based dairy cattle systems in southeast Brazil. J. Dairy Sci..

[31] Blagojevic B., Antic D., Ducic M., Buncic S. (2012). Visual cleanliness scores of cattle at slaughter and microbial loads on the hides and the carcases. Vet. Rec..

[32] Magrin L., Brscic M., Armato L., Contiero B., Cozzi G., Gottardo F. (2018). An overview of claw disorders at slaughter in finishing beef cattle reared in intensive indoor systems through a cross-sectional study. Prev. Vet. Med..

[33] Geenty K., Morris S.T. (2017). Guide to New Zealand Cattle Farming.

[34] Grandin T. (2010). Auditing animal welfare at slaughter plants. Meat Sci..

[35] Gibbs J. (2012). Fibre in New Zealand Pastures. VetScript.

[36] Bramley E., Costa N.D., Fulkerson W.J., Lean I.J. (2013). Associations between body condition, rumen fill, diarrhoea and lameness and ruminal acidosis in Australian dairy herds. N. Z. Vet. J..

[37] Blowey R.W., Weaver A.D. (2003). Color Atlas of Diseases and Disorders of Cattle.

[38] Langford F.M., Stott A.W. (2012). Culled early or culled late: Economic decisions and risks to welfare in dairy cows. Anim. Welf..

[39] Weik F., Archer J., Morris S., Garrick D., Hickson R. (2020). Relationship between body condition score and pregnancy rates following artificial insemination and subsequent natural mating in beef cows on commercial farms in New Zealand. N. Z. J. Anim. Sci. Prod.

[40] Burfeind O., Sepúlveda P., von Keyserlingk M.A.G., Weary D.M., Veira D.M., Heuwieser W. (2010). Technical note: Evaluation of a scoring system for rumen fill in dairy cows. J. Dairy Sci..

[41] Götze K., Crivellaro P., Pieper L., Engelhard T., Staufenbiel R. (2019). Assessment of rumen fill in dairy cows for evaluation of the individual feed intake in herd management. Tierarztl. Prax. Ausg. G Grosstiere Nutztiere.

[42] Hernández A., König S.E., Zúñiga J.J.R., Galina C.S., Berg C., Gonzales M.R., Villalobos A.D. (2017). Implementation of the welfare Quality^®^ protocol in dairy farms raised on extensive, semi-intensive and intensive systems in Costa Rica. J. Anim. Behav. Biometeorol..

[43] Sandøe P., Corr S.A., Lund T.B., Forkman B. (2019). Aggregating animal welfare indicators: Can it be done in a transparent and ethically robust way?. Anim. Welf..

[44] Petherick J.C., Doogan V.J., Holroyd R.G., Olsson P., Venus B.K. (2009). Quality of handling and holding yard environment, and beef cattle temperament: 1. Relationships with flight speed and fear of humans. Appl. Anim. Behav. Sci..

[45] Waynert D.F., Stookey J.M., Schwartzkopf-Genswein K.S., Watts J.M., Waltz C.S. (1999). The response of beef cattle to noise during handling. Appl. Anim. Behav. Sci..

[46] Phillips C.J.C. (1993). Cattle Behaviour.

[47] Hernandez A., Berg C., Eriksson S., Edstam L., Orihuela A., Leon H., Galina C. (2017). The welfare quality^®^ assessment protocol: How can it be adapted to family farming dual purpose cattle raised under extensive systems in tropical conditions?. Anim. Welf..

[48] Hemsworth P.H., Rice M., Karlen M.G., Calleja L., Barnett J.L., Nash J., Coleman G.J. (2011). Human–animal interactions at abattoirs: Relationships between handling and animal stress in sheep and cattle. Appl. Anim. Behav. Sci..

[49] Stafford K. (1997). Cattle Handling Skills.

[50] Smeaton D.C. (2003). Profitable Beef Production: A Guide to Beef Production in New Zealand.

[51] Grandin T. (2014). Handling facilities and restraint of extensively raised range cattle. Livestock Handling and Transport.

